# First report of the root-knot nematode, *Meloidogyne morocciensis* infecting peach in Southern Brazil

**DOI:** 10.21307/jofnem-2020-032

**Published:** 2020-04-09

**Authors:** W. R. Silva, C. P. Machaca-Calsin, C. B. Gomes

**Affiliations:** 1 Plant Protection Graduate Program, Federal University of Pelotas, RS 96010-900, Brazil; 2 Embrapa Temperate Agriculture, C.P. 403, Pelotas, RS 96001-970, Brazil

**Keywords:** Brazil, root-knot, *Prunus persica*, nematode

## Abstract

The peach tree (*Prunus persica*) is the third most cultivated temperate fruit species in Brazil. In August 2017, peach seedling plants showing symptoms of stunting and multiple galls in the roots were detected in the municipality of Pelotas, state of Rio Grande do Sul, Brazil. Samples of peach roots were collected and they were subsequently processed to obtain eggs and second-stage juveniles (J2), to determine the number of nematodes per gram of roots and to proceed with morphological studies. Additionally, individual females were extracted from the peach roots and submitted to *Meloidogyne* species identification by electrophoresis using α-esterase (Est) and malate dehydrogenase (Mdh) phenotypes and perineal pattern. The nematode population density in the samples was 283 eggs + J2s per gram of fresh roots. The polymorphism analysis revealed the A3N1 phenotype, typical to *Meloidogyne morocciensis*. Perineal patterns of females showed oval squared shapes, with moderately high to high dorsal arches, streaks widely separated, generally continuous, sometimes broken; the phasmids were separate by 29.3 μm (25.43-31.94 μm), similar to *M. arenaria* and *M. incognita*, as observed by Hammah and Hirschmann (1990). The second-stage juveniles had the following morphometric characters: L = 389.3 ± 3.8 (377.5-425.1) μm, stylet = 13.9 ± 0.2 (12.7-14.9) μm, DGO = 3.6 ± 0.1 (3.3-4.2) μm, tail length 47.5 ± 0.6 (45.3-48.9) μm, hyaline tail terminus = 14.1 ± 0.3 (12.5-17.0) μm, a = 25.6 ± 0.4 (23.8-28.8) μm and c = 8.6 ± 0.1 (8.0-9.3). The known SCAR marker obtained for *M. arenaria* is the same as that amplified for *M. morocciensis*. Under greenhouse conditions, peach plants seedlings inoculated with 1,000 eggs (Pi) with *M. morocciensis* were evaluated 28 days after inoculation compared to non-inoculated plants. The inoculated peach plants showed a mean of 338 galls per root system, and a nematode reproduction factor of 10.3. Besides this plants exhibited a reduction in fresh weight of shoots and roots compared to the non-inoculated plants. These results confirm *M. morocciensis* pathogenicity on *P. persica*.

The peach tree (*Prunus persica* (L.) Batsch) is the third most cultivated temperate fruit species in Brazil. In August 2017, peach seedlings showing symptoms of stunting and multiple galls on the roots (Fig. 1A) were detected in a nursery in Pelotas, Rio Grande do Sul, Brazil. Samples of peach roots were collected and subsequently processed (Hussey and Barker, 1973) to obtain eggs and second-stage juveniles (J2) to determine the number of nematodes per gram of roots and to for morphological studies (*n* = 20). Additionally, individual females (*n* = 20) were extracted from the peach roots and identified by electrophoresis using α-esterase (Est) and malate dehydrogenase (Mdh) phenotypes (Carneiro and Almeida, 2001) and perineal patterns (Taylor and Netscher, 1974). The nematode population density in the samples was 283 eggs and J2s per gram of fresh roots. Perineal patterns of females (Fig. 2B,C) showed oval squared shapes, with moderately high to high dorsal arches, striae widely separated, generally continuous, sometimes broken; the phasmids were 29.3 μm apart (25.4-31.9 μm), similar to *M. arenaria* (Neal, 1889) Chitwood, 1949 and *M. incognita* (Kofoid and White, 1919; Chitwood, 1949), as observed by Rammah and Hirschmann (1990). The polymorphism analysis revealed the A3N1 phenotype, Est A3 being the phenotype observed for α-esterase with three distinct bands (Rm = 1.11; 1.21; 1.32) (Fig. 1B) and Mdh N1 phenotype corresponding for malate dehydrogenase with only one band (Rm = 1.0) (Fig. 1C), typical to *Meloidogyne morocciensis* (Rammah and Hirschmann, 1990), according to Carneiro et al. (2008). Measurements and ratios of J2 were as follows in microns as means plus or minus the standard deviation with the range in parentheses (Fig. 2A): L = 389.3 ± 3.8 (377.5-425.1) μm, stylet length = 13.9 ± 0.2 (12.7-14.9) μm, DGO = 3.6 ± 0.1 (3.3-4.2) μm, tail length 47.5 ± 0.6 (45.3-48.9) μm, hyaline tail terminus length = 14.1 ± 0.3 (12.5-17.0) μm, a = 25.6 ± 0.4 (23.8-28.8) μm and c = 8.6 ± 0.1 (8.0-9.3). The known SCAR marker obtained for *M. arenaria* (Zijlstra et al., 2000) is the same as that amplified for *M. morocciensis* (Carneiro et al., 2008). Under greenhouse conditions, peach seedlings were inoculated with 1,000 eggs (Pi) from the original population of *M. morocciensis,* and non-inoculated plants were included. After 180 days, the plants were evaluated for the number of galls in the roots, and the final nematode population was also estimated (*Pf*). A mean of 338 galls per root system was observed and the nematode reproduction factor (RF = *Pf*/*Pi*) was 10.3. Inoculated plants exhibited a reduction in fresh weight of shoots and roots of 17 and 30%, compared to the non-inoculated plants, respectively. These results confirm the pathogenicity of *M. morocciensis* to peach. Some species of *Meloidogyne* have already been reported parasitizing peach, with an emphasis on *M. incognita* and *M. javanica* (Treub, 1885) Chitwood, 1949 (Carneiro et al., 1993). Although *M. morocciensis* has been detected in temperate fruit trees such as kiwi (*Actinidia deliciosa* (Chevalter) Liang and Ferguson) (Somavilla et al., 2011) and grapes (*Vitis vinifera* L.) (Divers et al., 2019) in Brazil, it is the first report of this species parasitizing *P. persica* in Brazil. This finding is of great importance for Brazilian agriculture, considering this nematode’s potential harm in the establishment of orchard and its effect on plant growth, fruit production, and the besides affecting plant growth, fruit production, and the longevity of peach trees.

**Figure 1:**
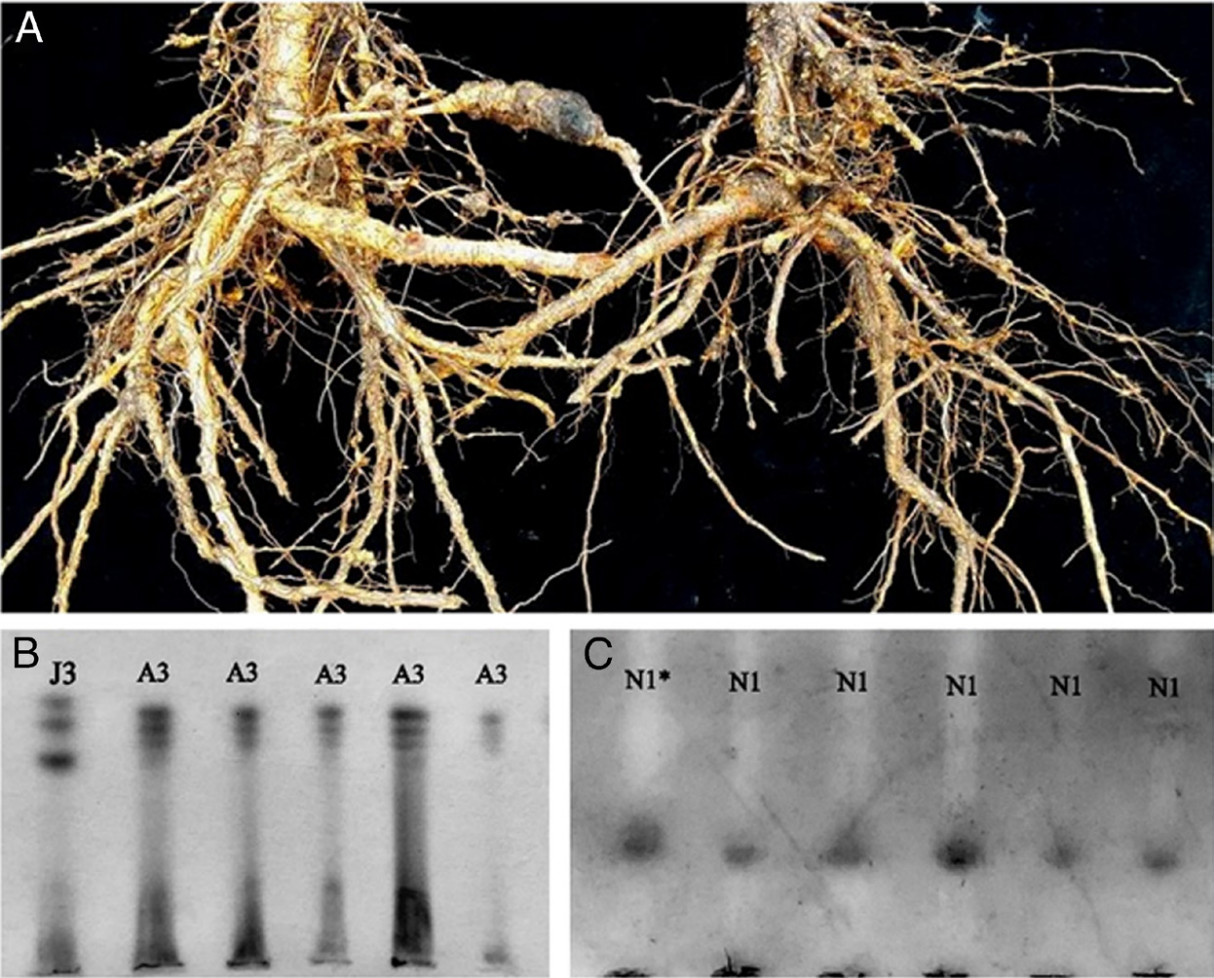
(A) Roots of peach plants parasitized by *Meloidogyne morocciensis* (Rammah and Hirschmann, 1990) with large and multiple galls. (B) Esterase phenotypes (A3) of *M. morocciensis* from peach and *M. javanica* (J3) as pattern reference. (C) Malate dehydrogenase phenotype (N1) of *M. morocciensis* from peach and *M. javanica* (N1*) as pattern reference. Photo (1.A) by Cristiano Bellé.

**Figure 2:**
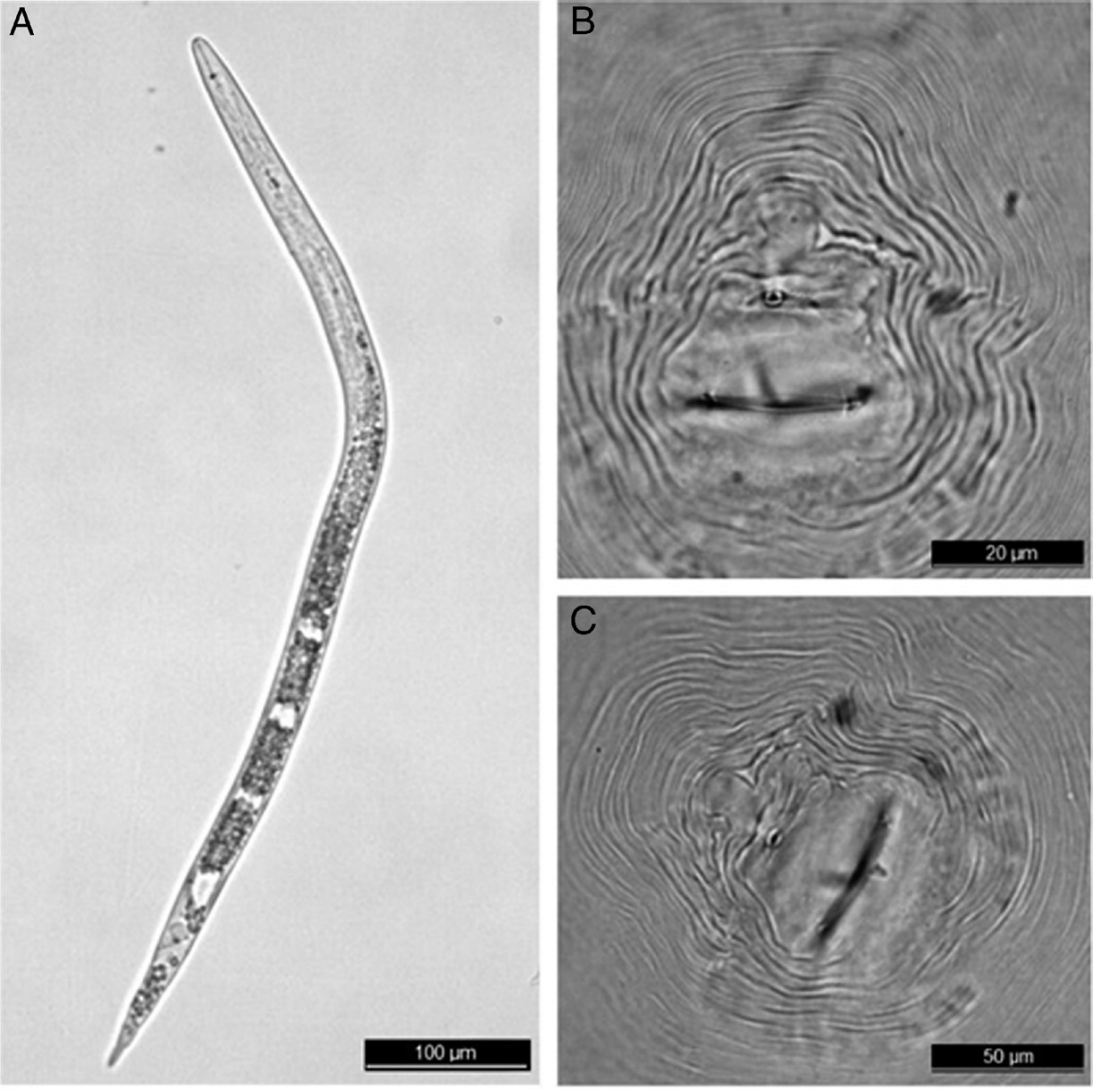
(A) Second-stage juveniles of *M. morocciensis*. (B-C) Perineal patterns of *M. morocciensis* adult females. (Scale bars: A: 100 μm; B: 20 μm; C: 50 μm).
